# Persistent Tunica Vasculosa Lentis in Full-Term Infants: A Report of Two Cases

**DOI:** 10.7759/cureus.11869

**Published:** 2020-12-03

**Authors:** Talaat Hamdi, Shamsher Ahmed Punekar, Mohammad Arif Mulla

**Affiliations:** 1 Ophthalmology, Jeddah University, Jeddah, SAU; 2 Ophthalmology, King Abdulaziz University Hospital, Jeddah, SAU

**Keywords:** lens, retina, tunica vasculosa lentis, retinopathy of prematurity, iris, eye anatomy, full term

## Abstract

This is a report of rare cases of full-term infants born with persistent tunica vasculosa lentis (TVL) with no retinopathy of prematurity (ROP) and no plus disease. This condition can be mistaken with iris vascular enlargement-associated plus disease, leading to unnecessary laser or intravitreal injections. The cases were treated with close observation, which resulted in complete resolution of the TVL. In conclusion, we encourage the diagnosis of TVL and careful monitoring of such cases before the intervention, as the condition may revert completely.

## Introduction

The development of human eye vasculature is a delicate balance between angiogenesis, vasculogenesis, and apoptosis. These mechanisms work in perfect harmony to provide the oxygen and nutrients which the growing tissue requires and halt any abnormal growth. A disturbance in this harmony can lead to many diseases, including retinopathy of prematurity (ROP).

ROP is a disease that affects all parts of the eye, as it was shown that it not only affects the retinal vasculature but also the fetal vasculature of the vitreous, which include the hyaloid vasculature and tunica vasculosa lentis (TVL) that supply the lens during its early development [1]. In the normal development of the human eye. TVL almost always regresses in a full-term baby and usually presents in preterm or ROP patients [2]. We present two rare cases that document a persistent TVL in full-term children with no ROP in Saudi Arabia.

## Case presentation

Case 1

This was a full-term female infant born at 39 weeks and five days of gestation by normal vaginal delivery, with a birth weight of 2.5 kgs, with a recorded Apgar score of 9 at one minute and 10 at five minutes. She was referred on the second day of life to the ophthalmology department, as the neonatologist noted an absent red reflex in the right eye of the baby.

Antenatal and perinatal histories were unremarkable. The ophthalmic examination conducted by the retina consultant revealed normal anterior and posterior segments in the left eye. The right eye was soft on digital examination and had mild conjunctival chemosis, minimal corneal haze, microhyphema, and a magenta-colored, tangled, whorled mass around the pupil extending onto the lens surface by a few millimeters, suggestive of persistent tunica vasculosa lentis. The pupil failed to dilate, so fundus evaluation was not possible, but a B-scan performed on the same day revealed anechoic vitreous and attached retina.

**Figure 1:**
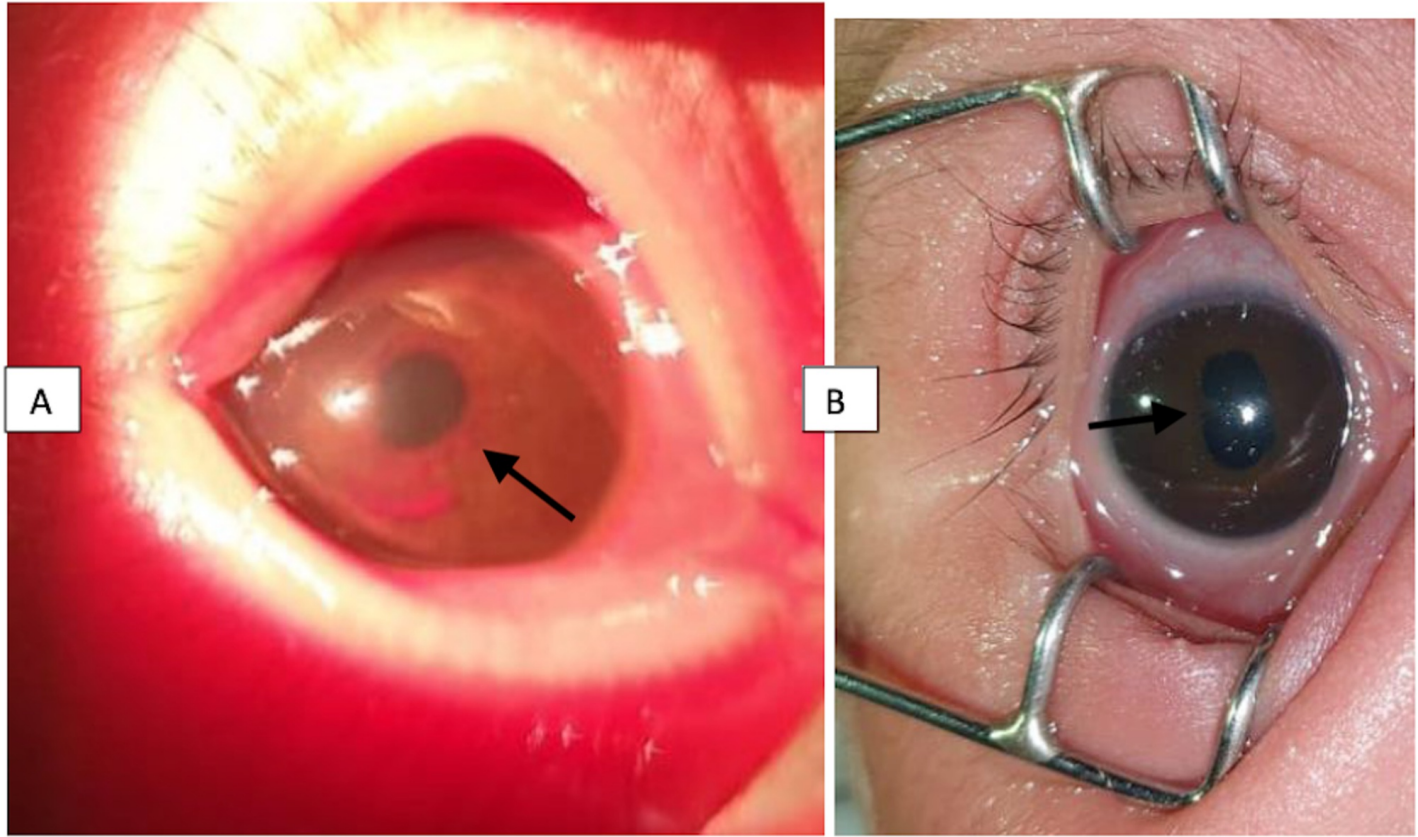
Colored photography of the right eye A) On the third day of life, showing mainly the microhyphema and a magenta-colored, tangled, whorled mass around the pupil extending onto the lens surface, as indicated by the arrow, suggestive of persistent tunica vasculosa lentis; B) After two weeks of conservative observation, showing the clearing of the TVL and mid-dilating pupil, as well as an area of posterior synechiae indicated by the arrow TVL: tunica vasculosa lentis

The child was followed up closely. On day 13 of her life, the examination of the right eye showed a clear cornea, microhyphema had disappeared, vascularity at the pupillary margin had markedly diminished, and the pupil was mid-dilated with dilating drops (Figure 1). Fundus examination performed at this stage revealed a normal retina in the right eye with no ROP signs nor plus disease.

Case 2

This was a 37 weeks' of gestation age female infant with a birth weight of 3.13 kgs and a recorded Apgar score of 8 at one minute and 9 at five minutes. Born by normal vaginal delivery with no significant antenatal nor post-natal history except that the mother was a type 1 diabetic. The cardiology team documented mild cardiomegaly that required no intervention. The patient was referred by the neonatal intensive care unit (NICU) team because of an absent red reflex on the seventh day of life.

The ophthalmologic examination conducted by a retina consultant showed a peripupillary, tangled vascular mass on the iris in both eyes suggestive of persistent TVL. The rest of the anterior segment examination was normal in both eyes, the intraocular pressure was taken by Schiotz tonometer showing normal ranges of 13-14 of both eyes. Unfortunately, the retinal view could not be achieved in both eyes due to non-dilating pupils. A B-scan done in both eyes, however, showed an anechoic vitreous and attached retina.

**Figure 2:**
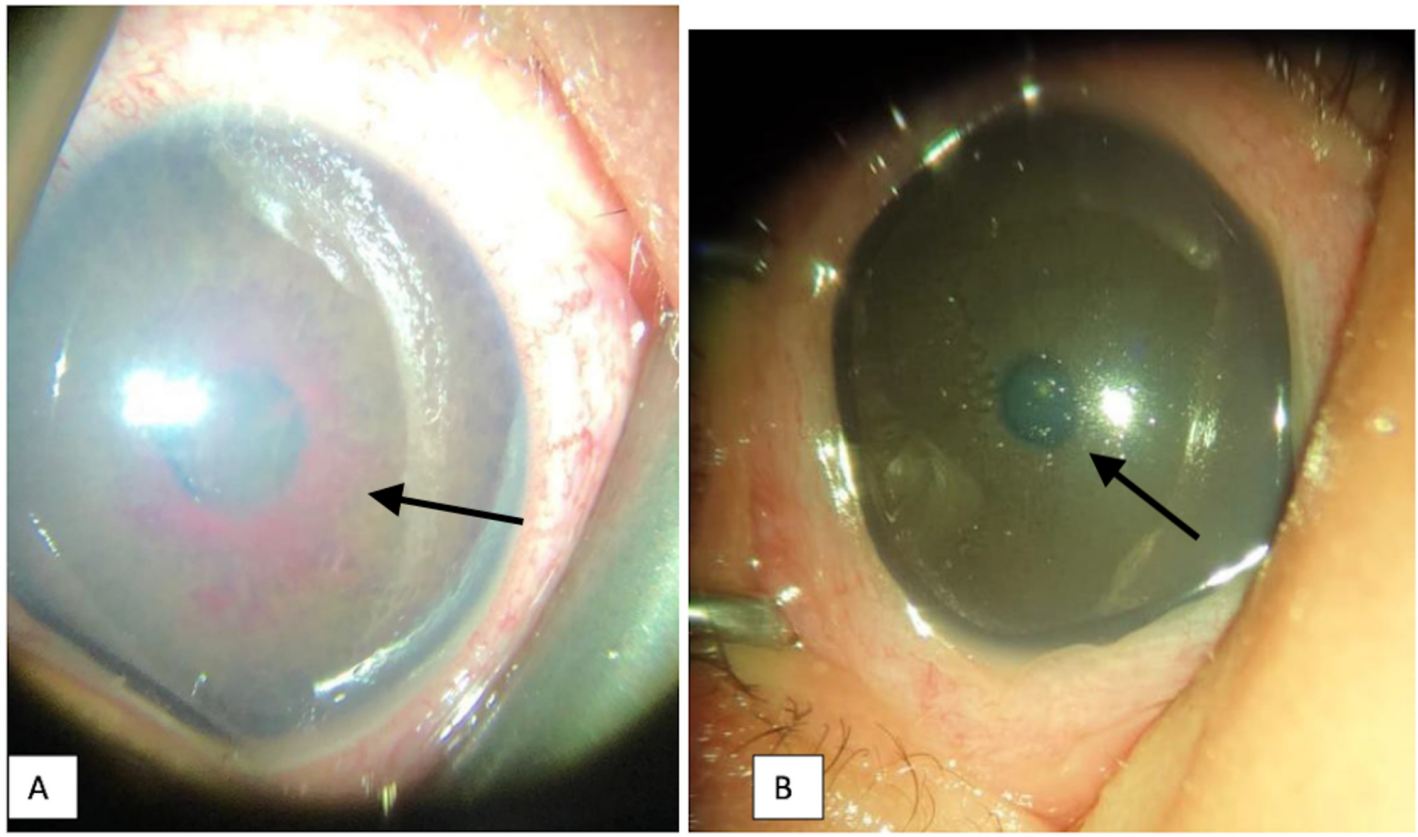
Colored photography of the left eye A) On the seventh day of life, showing a large whorled, red mass around the pupil extending onto the lens surface, indicated by the arrow, suggestive of persistent TVL; B) After one week of conservative observation, showing the clearing of the TVL TVL: tunica vasculosa lentis

Follow-up examinations after one week revealed complete disappearance of the TVL around the pupils, with limited dilatation around 4 mm as shown in Figure 2. In the second week, the pupils dilated further and the fundus examination was normal.

## Discussion

The development of the human eye starts as early as the formation of the optic pit at around 22 days of gestation, followed by further structural development to reach a fully mature eye. In normal eye anatomy, the TVL, which is part of the hyaloid vascular supply to the lens, disappears totally by term [3-4]. It can persist after birth in the case of an ROP patient, indicating a severe form [2]. The accurate prevalence of persistence TVL is not known, only a small number of studies reported such a condition in a full-term infant [5].

In our reported cases, persistent TVL was observed in full-term, healthy infants with no signs of ROP nor plus disease. Both cases had normal birth weights and normal deliveries. They only differed in family history, where the second case had a mother with type 1 diabetes. Only a few human neonate cases of persistent TVL in full-term babies are reported in the literature, many in animals [6-7]. The natural course of TVL is to disappear completely between 13 and 16 weeks of gestation [3]. When it endures after delivery, it gives rise to a difficult question of the proper course of management.

For persistent TVL associated with ROP, different modalities of treatment are considered. A conservative approach was promoted by Kumar et al. [8]. in an infant with stage 1, zone 3 ROP. Also, Favazza et al. showed that even in ROP rat models, persistent TVL regresses between 22 and 30 days of age and are undetectable by 64 days of age [9]. Intravitreal injections are another option of treatment and Higashiyama et al. reported to have used intravitreal injections in an ROP patient with TVL and poor dilatation, which regressed the TVL [10]. However, we do not fully know the long-term ocular and systemic consequences of anti-vascular endothelial growth factor use in preterm babies.

Since both of our patients were born after more than 37 weeks of gestation with normal deliveries and good birth weights, their persistent TVL being associated with underlying ROP was considered very unlikely. So a conservative method to observe the babies closely was decided. The absence of ROP could not be confirmed on the first examination, as pupils were not dilating because of TVL. But during the follow-up examination, the retina was found to be normal in eyes with persistent TVL and TVL regressed spontaneously without any intervention.

## Conclusions

We conclude that it is highly unlikely to have ROP in full-term babies with persistent TVL. So we recommend not to subject such babies to unnecessary intravitreal injections suspecting underlying ROP. Also, persistent TVL in these babies resolves spontaneously in a couple of weeks without any active intervention. Thus, we recommend close observation in mature babies with persistent TVL.
